# Vitamin D levels appear to be normal in Danish patients attending secondary care for low back pain and a weak positive correlation between serum level Vitamin D and Modic changes was demonstrated: a cross-sectional cohort study of consecutive patients with non-specific low back pain

**DOI:** 10.1186/1471-2474-14-78

**Published:** 2013-03-04

**Authors:** Jannick Vaaben Johansen, Claus Manniche, Per Kjaer

**Affiliations:** 1 Research Unit, Spine Centre of Southern Denmark, Part of Clinical Locomotion Network, Hospital Lillebaelt, Institute of Regional Health Services, University of Southern Denmark, Oestre Hougvej 55, Middelfart, DK-5500, Denmark; 2 Institute of Sports Science and Clinical Biomechanics, Part of Clinical Locomotion Network, University of Southern Denmark, Odense, Denmark

**Keywords:** Low back pain, Vitamin D, Modic changes

## Abstract

**Background:**

Hypovitaminosis D has previously been reported in both the general population, in people with chronic musculoskeletal pain, and in people with low back pain (LBP). Myopathy-related symptoms such as diffuse bone and muscle pain, weakness and paresthesia in the legs, have also been observed in people with non-specific LBP and associations with low levels of Vitamin D have been suggested. The objectives of this study were to investigate (1) Vitamin D levels in patients seeking care for LBP in a Danish out-patient secondary care setting, and (2) their possible relationship with myopathy-related symptoms, Body Mass Index (BMI), and Modic changes.

**Methods:**

A total of 152 consecutive patients with non-specific LBP participated in a cross-sectional study. Participants were recruited at The Spine Centre of Southern Denmark during springtime 2011. Individual serum levels of 25-Hydroxyvitamin-D were determined using Liquid Chromatography Tandem Mass Spectrometry (LC-MS/MS). Information about symptoms, height, and weight were collected from electronic questionnaires completed by the participants. All patients had an MRI from which Modic changes were identified. Correlations between Vitamin D level and pain, paresthesia, weakness in the legs, BMI or Modic changes were described using correlation coefficients and odds ratios obtained from logistic regression.

**Results:**

Two-thirds of the included patients with LBP had normal Vitamin D levels of >50 nmol/L. No correlations were seen between Vitamin D deficiency and gender, age, back pain intensity, leg pain intensity, and duration of pain. Statistically significant, but low, correlation coefficients were found between Vitamin D levels and BMI as well as Modic changes. Low Vitamin D levels and Modic changes were statistically significantly associated with an odds ratio of 0.30 (95% CI 0.12; 0.75) while weakness, paresthesia and widespread pain were not.

**Conclusions:**

In patients seeking care for low back pain in a Danish outpatient clinic, Vitamin D deficiency was not common. Whether patients who are overweight or who have Modic changes might represent subgroups of people for whom their LBP may be associated with Vitamin D levels, needs further investigation.

## Background

Internationally, there is consensus to define low back pain (LBP) as pain localised in the area below the costal margins and above the inferior gluteal folds [1]. LBP can be accompanied by leg pain (sciatica) or paresthesia in the legs. Forming a definitive patho-anatomical diagnosis in LBP is often difficult and is only possible in approximately 20% of patients attending primary care. The remaining 80% are often diagnosed with non-specific LBP [2]. The search for patho-anatomical causes of LBP is ongoing despite the fact that LBP is regarded as a multifactorial problem [3].

One line of research has investigated Modic changes as a potential cause of LBP. Dr. Modic and his group defined these as signal changes in the vertebral end plates seen on Magnetic Resonance Imaging (MRI) [4]. According to their definitions, the first stage is referred to as Type 1, which reflects hyper-vascularity in the vertebrae, probably as a result of inflammation. Type 2 consists of fatty replacements of the red bone marrow in the vertebrae. Modic changes seem to be associated with LBP and might constitute a patho-anatomical cause of LBP [5,6]. A systematic review by Jensen et al. showed a median prevalence of Modic changes of 43% in patients with non-specific LBP in contrast to a median prevalence of 6% in non-clinical populations [5], but the patho-genetic mechanisms underlying Modic changes are not completely understood [7]. It has been suggested that a possible cause of Modic changes is disc degeneration causing increased shear forces on the lumbar vertebral end plates leading to micro fractures. Modic changes could be either oedema-initialised by end plate micro fractures or an inflammatory response caused by pro-inflammatory chemicals seeping from the nucleus pulposus through such micro fractures [7].

Another line of research has drawn attention to Vitamin D and its relationship to the prevention of lifestyle-related diseases and inflammatory diseases [8,9]. Previous studies suggest that Vitamin D may be important in preventing Type 1-diabetes, cardiovascular heart disease, auto-immune diseases, depression and some cancers [8,10,11]. Vitamin D has also been suggested to have anti-inflammatory properties and has been shown to decrease pro-inflammatory cytokines and increase anti-inflammatory cytokines [9,12]. Several authors have suggested Vitamin D deficiency as a possible cause of chronic musculoskeletal pain [8,9].

Furthermore, Vitamin D has the ability to function as a hormone in the human body [9]. Its major function is to maintain calcium homeostasis. It is vital for development and maintenance of a healthy skeleton throughout life [9,13], and for maintaining optimal functioning of the muscles and nervous system [14,15].

Vitamin D deficiency is well documented worldwide [8,16], and is a common disorder in many regions, regardless of latitude [8,9,16-19]. Hypovitaminosis D has been reported in populations with several different types of chronic musculoskeletal pain such as osteoarthritis, rheumatoid arthritis, osteoporosis, soft tissue rheumatism, LBP, and arthralgia [9,17,19-23]. The reported musculoskeletal manifestations are diffuse muscle pain and weakness, muscle fatigue, paresthesia, arthralgia, and deep bone pain. These myopathic and arthralgic symptoms are non-specific and difficult to link to a specific diagnosis that may lead to other diagnoses such as fibromyalgia, polymyalgia and non-specific rheumatic diseases [14]. In clinical settings, adult patients with non-specific LBP often report accompanying diffuse musculoskeletal symptoms similar to those associated with Hypovitaminosis D as suggested above.

Therefore, we hypothesised that Danish people with non-specific LBP suffer from Vitamin D deficiency, as previously reported in both Egyptian and Saudi Arabian LBP populations [20,23], and that accompanying symptoms of diffuse pain in the back and legs, weakness, paresthesia, and Modic changes were related to Vitamin D deficiency.

Theoretically, there are two possible links between Hypovitaminosis D and LBP. Firstly, in patients with LBP, the diffuse pain in bone and muscle, weakness and paresthesia may be caused by Hypovitaminosis D. Secondly, Hypovitaminosis D could play a role in the development of Modic changes via the increased susceptibility to inflammation in the vertebral end plates [7]. Furthermore, low levels of Vitamin D cause increased serum parathyroid hormone leading to an increased bone turnover, which increases the risk of micro fractures in the vertebrae [24].

To our knowledge, no study exists on either the association between LBP and Vitamin D in northern latitude settings or possible associations between Vitamin D deficiency and the development of myopathy-related symptoms or Modic changes in LBP patients. Such knowledge may lead to new insights into the understanding and treatment of people suffering from LBP and the definition of a sub-group of patients with low levels of Vitamin D and specific clinical characteristics.

The objectives of this study were: (1) to describe the levels of serum Vitamin D in Danish patients with non-specific LBP, (2) to investigate how Vitamin D levels were related to the month of investigation, gender, age, BMI, duration of LBP, level of recent LBP, level of recent leg pain, number of pain sites, type of Modic changes, and (3) to examine possible associations between Vitamin D deficiency and Modic changes, muscle weakness, paresthesia and widespread pain.

## Methods

### Study design

The study was a cross-sectional cohort study of consecutive patients with non-specific LBP with or without leg pain seeking care in a Danish secondary out-patient hospital setting.

### Study population

Participants were recruited from patients referred to The Spine Centre of Southern Denmark from the end of March to the end of May 2011. Each year, The Spine Centre provides a comprehensive diagnostic evaluation for approximately 12,000 new patients with persistent cervical, thoracic and/or lumbar pain with or without radiation of symptoms into the limbs.

### Inclusion criteria

Patients between 18–65 years of age with non-specific LBP for more than 3 months, with or without leg pain radiation, were eligible for inclusion. The patients needed to have Danish or English language competency in order to complete the baseline ‘SpineData questionnaire’. The SpineData questionnaire includes a number of questions relevant to the current LBP episode, standardised measures of functioning (Roland Morris Disability Questionnaire), quality of life (EuroQOL-5D), general health (0–100 EuroQOL health thermometer), work situation, and the use of medication. Patients with other types of musculoskeletal pain and MRI (Magnetic Resonance Imaging) diagnosis of disc degeneration, disc bulging, or degenerative spondylolisthesis were accepted into the study.

### Exclusion criteria

Patients were excluded if they had contra-indications for lumbar spine MRI (ferromagnetic implants, claustrophobia etc.), or specific diagnosis of disc herniation, spinal stenosis, spondylolisthesis with spondylolysis, nerve root compression, fracture or metastases observable on MRI.

### Procedures

**Figure 1:**
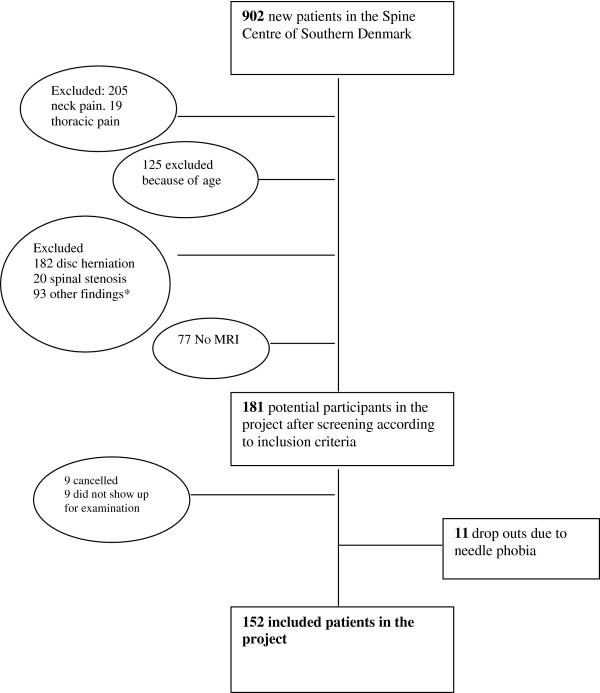
**Flowchart of LBP patient recruitment between March 21**^**th **^**and May 31**^**th **^**2011.** * Other findings: Arcolysis, nerveroot compression (lateral stenosis), fracture, possible metastasis.

The project leader (JVJ) screened all new referrals during the data collection period for patients fulfilling the inclusion criteria prior to their first visit at The Spine Centre. Patients who met the inclusion criteria were invited to participate in the study and, after having received oral and written information about the study, the participating patients gave their written consent. The Ethics Committee of Southern Denmark approved the study (Project ID: S-20110032). The Database Access Committee granted access to information on the participants collected in SpineData. The flow of participants is shown in Figure 1.

On arrival at The Spine Centre, each participant completed an electronic questionnaire that was stored in the SpineData database. For the analysis in this study, we used the variables listed and defined in Table 1.

**Table 1:** Descriptions and definitions of variables

**Variable**	**Provision of data and definition**
	**The variables marked **^**a **^**have been extracted from the SpineData database at The Spine Centre of Southern Denmark**
Vitamin D level	Measured by blood sample: Serum 25-Hydroxyvitamin-D specified in nmol/L
	Results divided into 3 groups: (1) normal Vitamin D level > 50 nmol/L, (2) mild Vitamin D deficiency between 25–50 nmol/L, and (3) severe/moderate Vitamin D deficiency < 25 nmol/L [14]
	Levels were measured in March, April and May 2011
Body mass index, BMI ^a^	Calculated from self-reported height (h) and weight from the database.
	Formula: BMI = mass (kg)/height^2^ (m)
Low Back Pain, LBP ^a^	Pain intensity, (0–10 numerical rating scales). Self-reported from the database.
	Patients were asked to enter: (1) back pain now, (2) worst back pain last two weeks and (3) average back pain last two weeks.
	LBP intensity is an average of the three entered scores [25]
Leg pain^a^	Pain intensity, (0–10 numerical rating scales). Self-reported from the database.
	Patients were asked to enter: (1) leg pain now, (2) worst leg pain last two weeks and (3) average leg pain last two weeks.
	Leg pain intensity is an average of the three entered scores [25].
Modic changes	Defined as signal changes in the vertebral end plates diagnosed on MRI [26] (Jensen, 2007). Data obtained from MRI descriptions.
	Modic changes were divided into groups: (1) No modic, (2) Modic Type 1, (3)
	Modic Type 2 and (4) Modic Types 1 and 2
Weakness in legs ^a^	Patients were asked about any weakness in the legs. Self-reported from the database (yes/no)
Paresthesia in legs ^a^	Patients were asked about any paresthesia in the legs. Self -reported from the database (yes/no)
Widespread pain ^a^	Patients reported their pain on a pain chart. In the database, the chart is divided into 46 different pain sites, which enables the database to calculate the number of pain regions

^a^ Patients completed an electronic version of the SpineData questionnaire which included a number of questions relevant to the current LBP episode: Pain (now, worst and average) the last two weeks, pain days per week, symptoms in the legs, previous back/neck pain, date of onset and the triggering factor of present pain, function (Roland Morris Disability Questionnaire), quality of life (EuroQOL-5D), general health (0–100 EuroQOL health thermometer, work situation and the use of medication). All data are self-reported.

Blood samples were taken on the same day by laboratory technicians at Lillebaelt Hospital, Middelfart. One sample was taken to analyse serum 25-Hydroxyvitamin-D, which has been suggested to be the best marker for measuring Vitamin D levels [9,18]. Blood samples were handled according to standard protocols and sent for analysis to the Department of Clinical Biochemistry in Vejle. Liquid Chromatography Tandem Mass Spectrometry (LC-MS/MS) was used for determining serum 25-Hydroxyvitamin-D.

### Definition of Vitamin D deficiency

In this study, we used the definition of Vitamin D deficiency suggested by Lips and the Danish recommendations from the National Food Institute [27,28]. Reference values were divided into four groups: (1) >50 nmol/L = normal, (2) 25–50 nmol/L = mild deficiency, (3) 12.5-25 nmol/L = moderat deficiency, and (4) <12.5 nmol/L = severe deficiency.

### Statistical analysis

A pre-hoc sample size calculation was performed for a single-group study and was based on the results from a previous study of chronic musculoskeletal pain in a Caucasian population and Hypovitaminosis D [19]. As we expected to have similar outcomes, the descriptive parameters (mean 35 nmol/L, standard deviation (SD) 18, 95% confidence interval (CI) 29–41) ) were used in the sample size calculation using an on-line calculator at Dimension Research [29]. That method recommended a requirement of 147 patients to ensure adequate power for this study.

Descriptive data for all variables included in the study were reported (means, SD and range for continuous variables; frequencies and proportions for categorical variables). Distributional graphs were inspected in order to determine the need for parametric or non-parametric statistical methods. Differences in distributions of descriptive data in the groups of Vitamin D deficiencies were cross-tabulated and tested using Fisher’s Exact Test. Correlations between Vitamin D levels and the variables of interest were visualised in scatter plots and further investigated using a Pearson correlation coefficient for continuous and normally distributed data and a Spearman correlation coefficient for categorical data and non-normally distributed data. Associations between low Vitamin D levels and clinical variables of interest were expressed as odds ratios with 95% CI obtained from logistic regression. For these analyses, the outcome variable (reduced Vitamin D) and explanatory variables (Modic changes yes/no, muscle weakness, paresthesia, and widespread pain if number of pain sites was > 5) were dichotomised.

The statistical software Stata/IC11.1 (StataCorp, Lakeway Drive, College Station, Texas USA) was used for the statistical analysis.

## Results

A total of 902 new referrals to The Spine Centre were screened for possible participation and 152 patients entered the study. Figure 1 shows the flow of patients and the reasons for non-participation. The participants included 100 women and 52 men aged between 19 and 64 years (mean 44.6 years). Other demographic information is given in Table 2.

**Table 2:** Characteristics of the LBP patients

**Descriptive statistics**	**Values**
**Age** (years) n = 152	44.6 ± 11.2 (19–64)
Mean ± SD (range)	
**Gender** n = 152	♀ 100 (66%)
Female ♀/male ♂ (%)	♂ 52 (34%)
**Body Mass Index, BMI** n = 138	26.0 ± 4.5 (18.9-38.9)
Mean ± SD (range)	
**LBP **^**a**^ n = 146	6.0 ± 2.0 (0.0-10)
Mean ± SD (range)	
**Leg pain **^**a**^ n = 146	4.1 ± 3.0 (0.0-9.7)
Mean ± SD (range)	
**Previous LBP episodes** n = 143	107 (74.8%)
Frequency (%)	
**Pain days per week:** (frequency (%)) n = 144	
Less than 1 day per week	2 (1.4%)
1-2 days per week	4 (2.8%)
3-4 days per week	13 (9.0%)
5-6 days per week	12 (8.3%)
Every day	113 (78.5%)
**Weakness in legs** n = 141	66 (46.8%)
Frequency (%)	
**Paresthesia in legs** n = 142	93 (65.5%)
Frequency (%)	
**Widespread pain** n = 143	
Frequency (%)	
≤ 5 pain areas	85 (59.4%)
> 5 pain areas	58 (40,6%)
**Vitamin D level** (nmol/L) n = 152	58.8 ± 26.2 (11–146)
Mean ± SD (range)	
**Modic changes** (frequency (%)) n = 152	
No Modic	112 (73.7%)
Modic Type 1	18 (11.8%)
Modic Type 2	13 (8.6%)
Modic Types 1 + 2	9 (5.9%)

^a^ Pain intensity on LBP and leg pain is an average (0–10 numerical rating scale) based on pain now (0–10), worst pain in the last 2 weeks (0–10) and average pain last 2 weeks (0–10) [25].

Only one patient had severe Vitamin D deficiency. Therefore, the categories ‘severe deficiency’ and ‘moderate deficiency’ were combined into one (*severe/moderate* deficiency < 25 nmol/L) for subsequent analysis. Most patients (99 (65.1%) of the 152 patients) had normal Vitamin D levels above 50 nmol/L, 36 (23.7%) had mild Vitamin D deficiency between 25–50 nmol/L, and 17 (11.2%) patients had moderate/severe deficiency < 25 nmol/L. The mean for Vitamin D levels for all patients was 58.8 nmol/L (SD 26.2).

**Figure 2:**
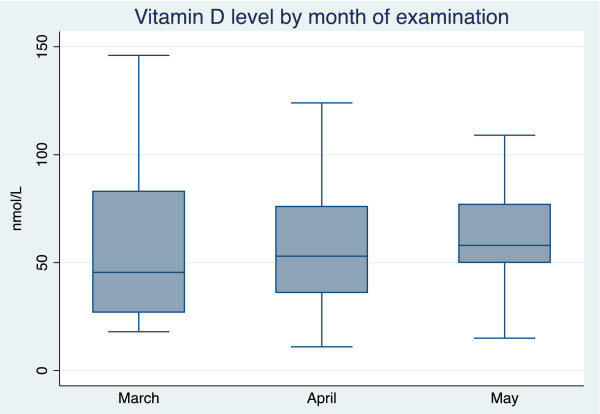
Vitamin D level by month (median, interquartile range).

Vitamin D levels increased during the 3-month investigation. In March, the mean Vitamin D levels in 22 new patients were 54.8 nmol/L ± 32.2 SD, range 18–146 nmol/L; in April, in 53 new patients were 56.5 nmol/L ± 28.5 SD, range 11–124 nmol/L; and in May, in 77 new patients were 61.6 nmol/L ± 22.4 SD, range 15–109 nmol/L; however, the increase was not statistically significant. The median and interquartile ranges are given in Figure 2.

**Figure 3:**
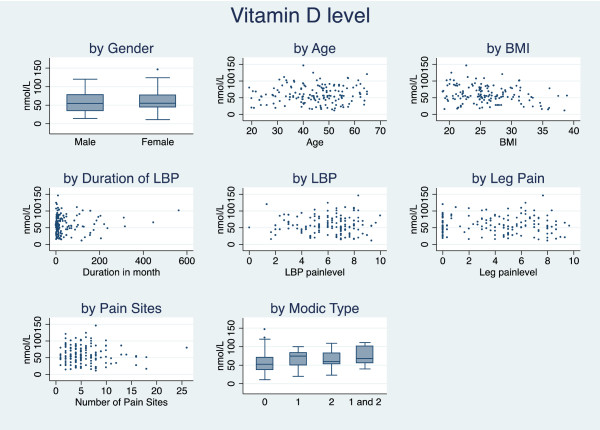
Relationships between Vitamin D levels and variables.

In total, 40 (26.3%) patients had Modic changes of which 18 (11.8%) had Type I, 13 (8.6%) had Type II and 9 (5.9%) had both Modic Types I and II. In the group of patients with normal Vitamin D levels (n = 99), 33 (33.3%) patients had Modic changes. In the group with a mild deficiency (n = 36), 5 (13.9%) patients had Modic changes and in the group with a moderate/severe deficiency (n = 17), 2 (11.8%) patients had Modic changes. The median values are shown in Figure 3. The remaining characteristics and descriptive statistics are listed in Table 2.

The correlation between Vitamin D levels and gender, age, BMI, duration of current episode, recent LBP, recent leg pain, number of pain regions and Modic changes are shown in Figure 3. Cross tabulating these variables, with the three groups of Vitamin D levels showed no statistically significant differences in distribution (data not shown). Only BMI and Modic changes were significantly correlated with Vitamin D level *(r*_*p*_ = −0.233, *p* = 0.006 and *r*_*s*_ = 0.221, *p* = 0.006 respectively, data not shown).

Only Modic changes were statistically significantly associated with low Vitamin D levels but in an inverse manner: Modic changes appeared to be protective of low Vitamin D level with an odds ratio of 0.30 (95%CI 0.12;0.75). Paresthesia, weakness and widespread pain were not associated with low Vitamin D (see Table 3).

**Table 3:** Association between clinical variables

**Variables**	**Odds ratio (CI 95%)**
Modic changes	0.30 (0.12;0.75)
Muscle weakness	1.08 (0.54;2.15)
Paresthesia	0.79 (0.38;1.62)
Widespread pain	1.10 (0.55;2.19)

(dichotomised) and Vitamin D deficiency (y/n).

## Discussion

This was the first study to investigate Vitamin D levels in patients living in northern latitudes who were seeking care in an outpatient clinic for their LBP. These patients had Vitamin D levels comparable to those in the general population in Denmark. Therefore, our hypothesis was not supported. Firstly, low levels of Vitamin D were not related to the clinical symptoms. Secondly, Modic changes were less common in people with low levels of Vitamin D. The current study could not support the existence of a specific subgroup that would benefit from treatment aimed at normalising Vitamin D levels.

The results of this study show that Vitamin D deficiency is not common in LBP patients, who are similar to those included in this study. The trend of an increase in Vitamin D levels from March until May is consistent with people being exposed to more sunlight during the northern Europe spring [16,18]. Vitamin D levels of LBP patients in the current study reflect those observed in the general Danish population. Mean Vitamin D levels of blood donors, who are supposed to be representative of at least the healthier segment of the general population in Denmark, have been reported to be 85 nmol/L (range: 26–163 nmol/L) in summer and 45 nmol/L (range: 13–128) in winter [18]. Previous studies by Glerup et al. have reported mean Vitamin D levels in healthy Danish women of 47.1 nmol/L (+/− 4.6) in a control group [30] whereas Brot et al. reported 53.5 nmol/L in a group of 280 middle-aged women regularly exposed to sun and not taking vitamin supplements [31].

A sufficient level of Vitamin D in serum is continuously discussed in the literature and it is still subject to debate [9,10,18]. Previous studies that reported low levels of Vitamin D in populations with LBP used different reference criteria for normal values >100 nmol/L (>40 ng/ml) [23] and > 22.5 nmol/L [20], which makes comparison between studies difficult. Haroon et al. investigated 231 consecutive patients in general clinics using a reference criterion for a normal value of >53 nmol/L [21]. Of the 231 patients, 8 had backache, 6 (75%) of whom had Vitamin D deficiency. Our results differ from the findings of Haroon et al., but comparison is difficult since only eight patients had backache.

Multiple factors affect the ability to synthesise Vitamin D, such as latitude, skin pigmentation, clothing, exposure to sunlight, obesity, malnutrition, and protein deficiency [13,16,24]. These factors complicate the direct comparison of Egyptian and Saudi Arabian people with people from countries in northern latitudes. Individual thresholds exist for the need for certain minerals and vitamins in humans. It is possible that thresholds differ across ethnic groups for the development of musculoskeletal symptoms due to a deficiency in certain minerals and vitamins, including Vitamin D.

The available methods for analysing 25-OH-D show significant differences in levels depending on which method is used [32,33]. Lips et al. compared three different methods of measuring Vitamin D levels [33]. A difference of 80% between Competitive Protein Binding (CPB) assay was seen when compared with High-Performance Liquid Chromatography (HPLC), while Radioimmunoassay (RIA) gave intermediate values [33]. The LC-MS/MS method that we used in this study is reported to be the most reliable method [32]. Despite this, variability in results between reporting laboratories can be up to 15% [32].

Our results did not support our hypothesis, and are in contrast to the findings in several previous studies on both chronic musculoskeletal pain and LBP, which suggests there might be several musculoskeletal symptoms related to low levels of Vitamin D [17,19-23]. However, the lack of association between LBP and low levels of Vitamin D has been shown previously: Heidari et al. found significantly lower Vitamin D levels in patients with non-specific skeletal pain, but, similar to our findings, not in the group of back pain patients [22]. In another study, Lotfi et al. found no associations between Vitamin D levels and back pain intensity or duration of LBP [23].

The lack of association we found between widespread pain and low Vitamin D levels is in contrast to the findings of McBeth et al. who reported that people with chronic widespread musculoskeletal pain have a 50% increased risk of low levels of Vitamin D [17].

We found no association between Vitamin D levels and myopathy-related symptoms. This could be due to either a small group of patients in the moderate/severe-group (n = 17 (11%) and only one with severe Vitamin D deficiency), or the fact that the symptoms were self-reported and not objectively measured. Glerup et al. found that most myopathy-related symptoms are present at Vitamin D levels below 25 nmol/L and that the first symptom most often reported is fatigue and not pain or paresthesia [14], which could account for the lack of association in our study.

Our finding of a negative correlation between Vitamin D deficiency and high BMI has previously been reported and can be explained by a storage of Vitamin D in adipose tissue [9,16,34]. Further investigations aimed at examining whether there is a relation between LBP, high BMI and levels of Vitamin D could be interesting in the search for relevant subgroups within LBP patients.

Unexpectedly, Modic changes were more common in people with normal levels of Vitamin D compared with those with low levels. According to our theoretical framework, people with Modic changes were expected to have low levels of Vitamin D due to an increased susceptibility to inflammation [12] and/or to micro-fractures in the vertebrae due to increased levels of parathyroid hormone [24]. Only serum 25-OH-D were measured in this study. Therefore, it is not possible to draw conclusions about increased bone turnover in the investigated group of LBP patients, although levels of < 50 nmol/L have been shown to cause an increased level of parathyroid hormone [24]. Our initial theoretical framework failed to explain the possible relationship between Vitamin D and Modic changes. Perhaps, the increased risk of micro-fractures related to cortical bone due to hyperparathyroidism [24] does not apply to the vertebra end plates, which mainly consist of hyalin cartilage, and therefore the significant finding was perhaps simply a random and sample-specific artifact.

The mechanisms in the development of Modic changes are still not sufficiently known. Current significant findings suggest that there could be a link between Vitamin D and Modic changes perhaps related to inflammation. Further studies are needed on the subject.

The strengths of this study are that participants were recruited from consecutive patients, standardised institutional methods were used for collecting self-reported data, and blood samples were taken on the same day as filling in the self-report questionnaires. However, while the included patients might be representative of non-specific LBP patients at The Spine Center of Southern Denmark, it is not known how comparable they are with patients in other settings.

Our study does not support an identifiable sub-group of people with LBP and Vitamin D deficiency who could benefit from treatment with Vitamin D. This is in line with a Cochrane review that concludes that there is not sufficient evidence to support treatment of chronic pain with Vitamin D [35]. The study had a cross-sectional design. A randomised control trial is needed to further investigate whether people with non-specific LBP would benefit from Vitamin D treatment. Furthermore, the design does not allow us to make conclusions regarding adequate thresholds of 25 (OH)D in non-specific LPB patients. This is still a relevant research question.

LBP is a common disorder throughout the world with two-thirds of all adults experiencing back pain at some time in their life [36]. The group of non-specific LBP patients is a heterogeneous group with multiple factors contributing to the symptoms experienced by the individual patient. Many different biological and psychosocial factors can contribute to LBP symptoms [36,37], and therefore, it is difficult to isolate a single reason for non-specific or chronic LBP in a given individual. Perhaps the underlying mechanisms of non-specific LBP differ in different regions of the world and perhaps these mechanisms differ across ethnic groups.

## Conclusions

The results of this study indicated that a group of Danish people with non-specific LBP did not have Vitamin D deficiency, and no relationships were found between those with Vitamin D deficiency and myopathy-related symptoms of weakness and paresthesia in the legs, back pain or leg pain intensity. LBP patients with Vitamin D deficiency did not have different clinical characteristics compared with LBP patients with normal Vitamin D levels. The observation that LBP patients with Modic changes had relatively higher Vitamin D levels warrants further study.

## Abbreviations

LBP: Low back pain; BMI: Body mass index; LC-MS/MS: Liquid chromatography tandem mass spectrometry; MRI: Magnetic resonance imaging.

## Competing interests

There are no competing interests, either financial or non-financial.

## Authors’ contributions

JVJ designed the project, carried out the recruitment of patients, coordinated the collection of the data, assisted in the interpretation of the data and drafted the manuscript. CM participated in the design of the project, assisted in the coordination and the interpretation of the data. PK participated in the design of the project, performed the statistical analysis, carried out the interpretation of the data and helped draft the manuscript. All authors read and approved the final manuscript.

## Pre-publication history

The pre-publication history for this paper can be accessed here:

http://www.biomedcentral.com/1471-2474/14/78/prepub
